# Chatbot-aided product purchases among Generation Z: the role of personality traits

**DOI:** 10.3389/fpsyg.2025.1454197

**Published:** 2025-08-29

**Authors:** Jinrong Liu, Jialiang Chen

**Affiliations:** ^1^Department of Physical Education, University of Shanghai for Science and Technology, Shanghai, China; ^2^Shanghai University of Sport, Shanghai, China

**Keywords:** product recommendation chatbots, Generation Z, Big Five personality traits, source characteristics of information, SEM-ANN-NCA

## Abstract

**Introduction:**

The rapid integration of machine learning has positioned product recommendation chatbots as essential tools in the e-commerce landscape, shaping how consumers engage and make purchasing decisions. Generation Z, as a tech-savvy and AI-adept demographic, plays a central role in this transformation. While prior studies have examined chatbot-consumer interactions, limited research has explored how both personality traits and information source characteristics jointly influence purchase intentions.

**Methods:**

This study develops an integrative framework to assess how the Big Five personality traits—extraversion, agreeableness, conscientiousness, neuroticism, and openness—and key chatbot features—expertise, interactivity, trustworthiness, and customization—affect Generation Z’s willingness to purchase chatbot-recommended products. The moderating role of personal innovativeness is also examined. Data were collected from 480 Generation Z chatbot users in China through an online survey and analyzed using structural equation modeling (SEM), artificial neural networks (ANN), and necessary condition analysis (NCA).

**Results:**

Results indicate that extraversion, agreeableness, openness, expertise, interactivity, and customization significantly influence purchase intention. Moreover, personal innovativeness positively moderates the effect of extraversion on purchase intention.

**Discussion:**

These findings contribute to the literature by bridging personality psychology and human–AI interaction and offer practical insights for enhancing chatbot effectiveness in e-commerce.

## 1 Introduction

With the rapid advancement of artificial intelligence (AI) technologies, e-commerce firms are increasingly integrating conversational agents into their digital touchpoints to deliver highly personalized and interactive marketing experiences (Peng et al., 2023; Wang, 2023). Product-recommendation chatbots utilize natural language processing and machine learning algorithms to curate item assortments tailored to individual consumer preferences, thereby enhancing perceived utility and shopping satisfaction (Jin and Eastin, 2023). Real-world implementations exemplify this trend. For example, Lazada, a leading Southeast Asian platform, has launched LazzieChat, a GPT-powered assistant that provides real-time personalized suggestions. Similarly, Taobao has developed Ali Xiaomi, a conversational interface that promotes user engagement through socially oriented dialogs (Yin and Qiu, 2021). Empirical evidence suggests that such emotionally enriched human–AI interactions positively affect consumer affect and purchasing intentions (Wang C. et al., 2023; Wang L. et al., 2023; Wang X. et al., 2023).

In this context, scholarly attention has increasingly focused on how consumers interact with chatbots. Prior studies have demonstrated that variations in chatbot design—such as language style, anthropomorphic cues, and emoticon use—evoke different consumer responses (Li and Shin, 2023; Li and Wang, 2023; Lu et al., 2024). However, users’ psychological profiles, socioeconomic backgrounds, and cultural traits also play a critical role in shaping human–computer interaction (Arpaci et al., 2022). While personality traits have been shown to influence engagement with AI-based systems (Arpaci and Kocadag Unver, 2020), their role in chatbot-mediated product recommendations remains insufficiently understood. Simultaneously, chatbots serve as information sources whose perceived characteristics—such as expertise, trustworthiness, and interactivity—can directly influence consumer evaluations and behavioral intentions (Han, 2021; Shin et al., 2023). Thus, considering both personality traits and information-source attributes may provide deeper insights into decision-making mechanisms in AI-mediated commerce.

Despite these insights, two major research gaps remain. First, prior work has tended to examine personality and information-source factors in isolation, failing to explore how they might interactively shape consumer intention. Second, most studies rely on linear modeling techniques that may not adequately capture the complex, nonlinear relationships among psychological and technological variables. These limitations hinder our understanding of how Generation Z responds to chatbot recommendations in dynamic digital environments.

To address these gaps, this study proposes the following research questions: (1) How do personality traits shape Generation Z consumers’ intentions to purchase chatbot-recommended products? (2) How do different chatbot information attributes affect purchase intention? (3) Does personal innovativeness moderate the effect of personality traits on purchase decisions? (4) How do different analytic approaches—linear, nonlinear, and necessity-based—converge or diverge in their interpretations of these relationships?

To answer these questions, this study adopts an integrated methodological framework combining partial least squares structural equation modeling (PLS-SEM), artificial neural networks (ANN), and necessary condition analysis (NCA). This multi-method approach enables us to capture both linear and nonlinear relationships, as well as necessary conditions for specific outcomes, thereby offering a more comprehensive understanding of the mechanisms driving Generation Z’s behavioral responses to chatbot product recommendations.

## 2 Research design

In the initial phase of this study, partial least squares structural equation modeling (PLS-SEM) was employed to examine the linear relationships among latent variables and to validate the hypothesized conceptual model. As a prominent variance-based technique within the SEM family, PLS-SEM integrates features of principal component analysis and multiple regression, offering flexibility in handling complex, multi-path models with relatively small sample sizes and non-normally distributed data (Lew et al., 2020). Given the exploratory nature of the present study and the presence of both reflective and formative constructs, PLS-SEM was selected as the primary tool for testing the theoretical pathways linking the Big Five personality traits, chatbot information-source characteristics, and purchase intention.

To complement the linear perspective of PLS-SEM and explore potential nonlinear patterns in the data, artificial neural network (ANN) analysis was incorporated in the second phase. As a data-driven computational technique, ANN is well-suited to model high-order interactions and nonlinear dependencies without imposing distributional assumptions (Lo et al., 2022). In this study, the latent scores extracted from PLS-SEM were used as input features for a three-layer feedforward ANN comprising an input layer (corresponding to the extracted components), a hidden layer, and a single-node output layer predicting purchase intention. The hidden layer adopted the ReLU activation function, while the output layer utilized a Sigmoid function to produce probabilistic outcomes. The network was trained using the Adam optimizer with a learning rate of 0.01, batch size of 32, and early stopping based on validation loss. Ten-fold cross-validation was performed to ensure generalizability, and model fit was evaluated using mean squared error (MSE) and prediction accuracy. In addition, a permutation-based sensitivity analysis was conducted to derive the relative importance of each predictor, providing a ranking of the most influential factors in driving behavioral intention.

Although the combined use of PLS-SEM and ANN allowed for the identification of both linear and nonlinear associations, these approaches do not evaluate whether certain variables constitute indispensable prerequisites for behavioral outcomes. Therefore, in the third analytical phase, necessary condition analysis (NCA) was implemented to determine whether specific antecedents functioned as non-compensatory constraints for the occurrence of high purchase intention (Richter et al., 2020). NCA operates under the logic of necessity rather than sufficiency: it posits that if a necessary condition is not met, the desired outcome cannot occur, regardless of the levels of other predictors. The analysis was conducted using the CE-FDH (free disposal hull) method in RStudio, and significance testing was performed using 10,000-fold permutation sampling. By identifying minimum thresholds that must be exceeded for the outcome to manifest, NCA provides a complementary diagnostic lens that extends beyond correlational inference.

**Figure 1:**
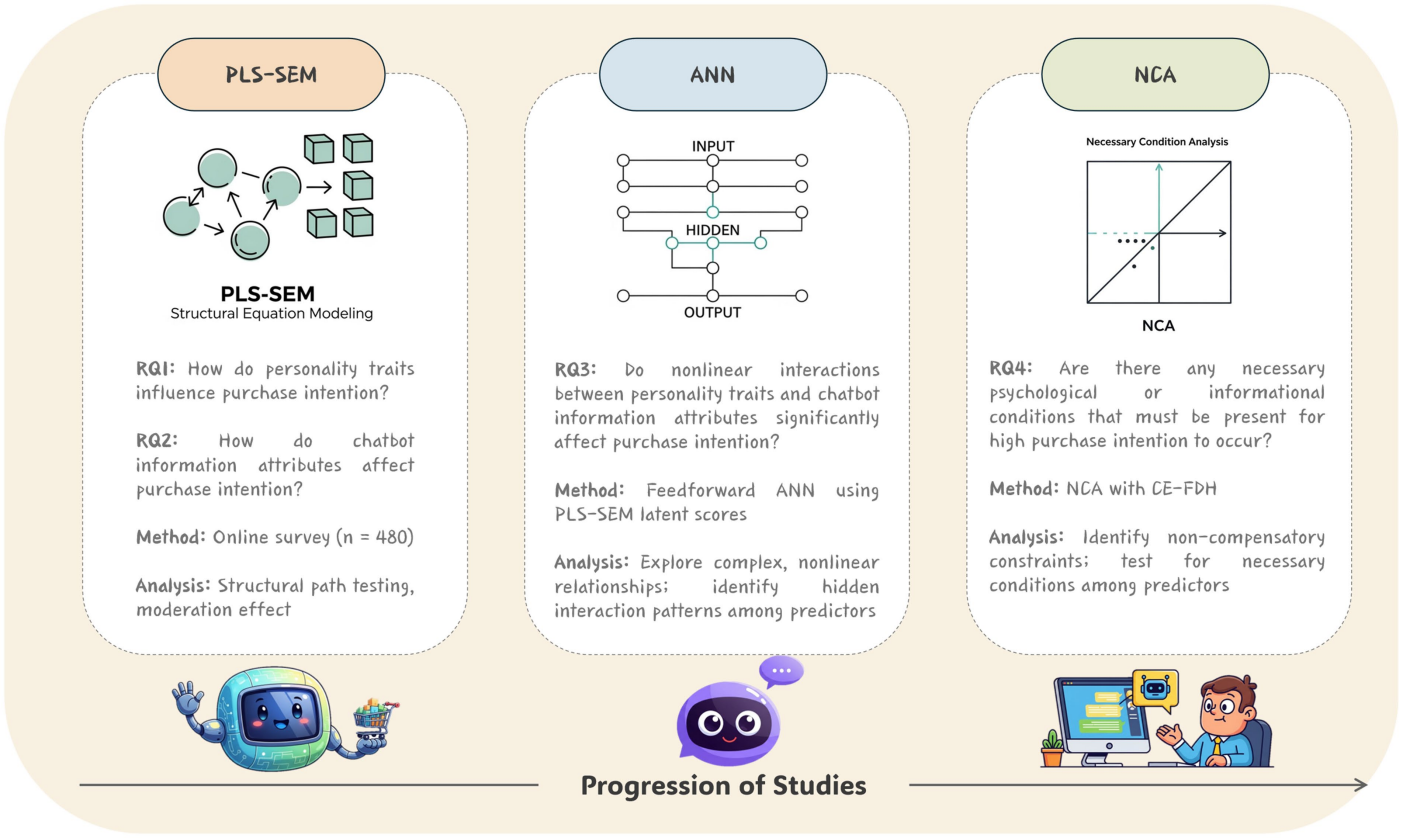
Analytical framework combining PLS-SEM, ANN, and NCA.

Taken together, the integration of PLS-SEM, ANN, and NCA constitutes a comprehensive, triangulated analytical framework that captures linear causality, nonlinear complexity, and asymmetrical necessity (see Figure 1). This hybrid approach enables a deeper understanding of the multidimensional mechanisms through which personality traits and information source characteristic jointly shape Generation Z’s product-purchase decisions in AI-mediated retail environments. Beyond statistical robustness, this design also aligns with theoretical pluralism by combining hypothesis-driven testing with data-centric exploration and constraint-based reasoning.

## 3 Conceptual background and hypotheses development

As e-commerce platforms increasingly embed artificial intelligence technologies into their service architecture, product-recommendation chatbots have become a crucial tool for enhancing the consumer experience (Rahevar and Darji, 2024). These AI-driven agents not only offer personalized product suggestions but also facilitate highly interactive, human-like dialogs, thereby transforming conventional online shopping into a more engaging and socially enriched experience (Chen et al., 2025). Among diverse consumer segments, Generation Z—digital natives born between 1997 and 2012—demonstrates a particularly high level of openness to AI technologies and a pronounced reliance on algorithmic support in decision-making processes (Bunea et al., 2024). To understand how this cohort responds to chatbot-based product recommendations, it is essential to construct a theoretical framework that encompasses both individual psychological dispositions and users’ perceptions of chatbot characteristics.

To this end, the present study draws upon two classical theoretical perspectives—namely, the Big Five personality trait model and source credibility theory—to explain the formation of purchase intention in AI-mediated retail settings. The Big Five model categorizes personality across five dimensions: extraversion, agreeableness, conscientiousness, neuroticism, and openness to experience. These traits have demonstrated robust predictive validity across a variety of digital behaviors, including technology adoption, trust in automated systems, and responsiveness to recommendation algorithms (Seyfi et al., 2025). Prior studies have indicated that although Generation Z is generally receptive to technological innovation, their personality profiles differ significantly, potentially leading to varied patterns of interaction with AI agents (Ding et al., 2025). For instance, individuals with high openness are more likely to embrace novel chatbot interfaces, while those with high conscientiousness tend to pay closer attention to the quality of the information provided (Kovbasiuk et al., 2025).

At the same time, source credibility theory posits that perceived expertise, trustworthiness, and interactivity are key factors influencing how users evaluate information sources (Sardar et al., 2024). In the context of AI chatbots, these attributes are not only embedded in system design but also subjectively interpreted by users during the interaction process (Anbalagan et al., 2025). When a chatbot is perceived as competent, reliable, and engaging, its recommendations are more likely to be viewed as persuasive and actionable (Kim and Priluck, 2025). For Generation Z, characterized by high digital literacy and extensive exposure to mediated content, these source-level cues may be especially influential in shaping behavioral outcomes (Ding et al., 2025).

The integration of these two theoretical perspectives contributes to a more comprehensive understanding of how purchase intentions are formed in chatbot-mediated contexts. The Big Five framework elucidates stable intrapersonal differences in psychological predisposition, while source credibility theory reveals how users cognitively and affectively respond to external system cues during interaction. This integrative framework allows for the simultaneous modeling of internal psychological drivers and external perceptual influences on consumer decision-making, which is particularly well-suited for uncovering the dual-layered mechanisms by which Generation Z users engage with AI-based recommendation agents. As such, the integration of these theories aligns with the complex and interactive nature of technology-mediated consumption and directly addresses this study’s dual emphasis on personality-driven variability and source-driven persuasion.

### 3.1 Big five personality traits

Individual cognition and behavior may be shaped by various factors, including personal experiences, living environment, and educational attainment (Baumert et al., 2017; Salmony and Kanbach, 2022). Personality is widely regarded as a foundational psychological construct that influences how individuals perceive, feel, and act (Sadeq et al., 2018; Louwen et al., 2023). It is jointly determined by genetic predispositions and socio-cultural factors. While many personality traits have a hereditary basis, social environments contribute significantly to their formation, guiding individuals toward similar personality structures (Hörz-Sagstetter et al., 2021). Personality encapsulates a distinct configuration of thoughts, emotions, and behaviors that distinguishes one individual from another, reflecting consistent and coherent psychological attributes (Boudreaux, 2016). Individual personality traits have been shown to predict and explain specific behavioral tendencies (Zweig and Webster, 2003; Busseri and Erb, 2024), including technology adoption behaviors (Liu et al., 2024). Examining new technology usage from the perspective of personality traits offers deeper insights into the psychological mechanisms underlying individual acceptance. Prior research indicates that personality traits significantly affect individuals’ adoption of internet platforms (Svendsen et al., 2013), social media (Liu and Campbell, 2017), metaverse applications (Kumar et al., 2024), and artificial intelligence technologies (Riedl, 2022).

Personality is a multifaceted system encompassing temperament, character, cognitive styles, and self-regulatory processes. It is marked by distinctiveness, consistency, comprehensiveness, and adaptability (Haslam et al., 2004; Cuartero and Tur, 2021). Trait theory posits that personality consists of a constellation of traits—stable, cross-situational patterns of behavior and thought (Bleidorn et al., 2016). Traits are defined as enduring dispositions with moderate temporal stability, capturing individual preferences and behavioral tendencies (Fajkowska, 2017; Hopwood, 2025). The big five personality traits—extraversion, agreeableness, conscientiousness, neuroticism, and openness—are the most widely accepted framework in contemporary psychology (Lynn, 2021).

Extraversion describes individuals’ social energy and activity levels. It embodies enthusiasm, sociability, and assertiveness, reflecting the extent of engagement in interpersonal interactions (Buecker et al., 2020). Extraverts typically thrive in group settings and enjoy engaging in new experiences. In contrast, introverts prefer solitude and derive energy from introspection (Eid et al., 2003). Due to their curiosity and exploratory tendencies, extraverts are more likely to engage with chatbots and respond positively to product recommendations.

Agreeableness captures traits related to altruism, empathy, and cooperation. Individuals high in agreeableness are typically considerate, trusting, and inclined toward social harmony (Wilmot and Ones, 2022). In contrast, those low in agreeableness may exhibit greater skepticism and competitiveness (Stavrova et al., 2022). Agreeable individuals tend to respond positively to personalized experiences; hence, chatbot recommendations that align with their interpersonal orientation may increase purchase intentions.

Conscientiousness reflects goal-directed behavior and self-discipline. It is associated with organization, responsibility, and persistence (Buelow and Cayton, 2020). High-conscientiousness individuals are more likely to engage in planned, deliberate decision-making. They may place greater trust in reliable and well-structured chatbot systems, perceiving them as extensions of efficient service. Conversely, low-conscientiousness individuals may prefer spontaneity and demonstrate less concern for rule-based interactions (Fleischmann et al., 2023).

Neuroticism refers to emotional instability and sensitivity to stress (Asaoka et al., 2020). Individuals scoring high in neuroticism are more prone to negative emotions, such as anxiety and mood swings, while emotionally stable individuals tend to be calm and resilient (Lee and Bottomley, 2021). In a consumer context, neuroticism may influence the emotional reactions to chatbot interactions and increase the likelihood of impulse purchases under stress or emotional arousal.

Openness denotes intellectual curiosity, creativity, and a preference for novelty (Tucaković and Nedeljković, 2022). Open individuals are more receptive to new experiences and are more likely to explore unfamiliar options (Gil de Zúñiga et al., 2017). Consequently, they may be more engaged with innovative chatbot functionalities and willing to accept AI-generated product recommendations. These individuals are also more adept at integrating new information into their decision-making processes.

Therefore, the following hypothesis is proposed:

*H1:* Extraversion has a significant positive impact on Generation Z’s intention to purchase products recommended by product-recommendation chatbots.

*H2:* Agreeableness has a significant positive impact on Generation Z’s intention to purchase products recommended by product-recommendation chatbots.

*H3:* Conscientiousness has a significant positive impact on Generation Z’s intention to purchase products recommended by product-recommendation chatbots.

*H4:* Neuroticism has a significant positive impact on Generation Z’s intention to purchase products recommended by product-recommendation chatbots.

*H5:* Openness has a significant positive impact on Generation Z’s intention to purchase products recommended by product-recommendation chatbots.

### 3.2 Information source characteristics

In societal contexts, information encompasses a wide array of content that serves as a ubiquitous medium, linking individuals with knowledge about people, events, and objects. Within the sphere of electronic commerce, information plays a critical role (Zhong and Han, 2023). As the vehicle for disseminating content, the information source determines both the quality and substance of that content, rendering its role indispensable. The trustworthiness and effectiveness of information sources have been extensively examined in theoretical models, which explain how various source attributes influence audience attitudes and behaviors (Kelman, 2017). Through processes such as production, processing, storage, and dissemination, information reaches its recipients and exerts persuasive effects—an influence regulated largely by the characteristics of the information source. These persuasive effects shape recipients’ attitudes, cognitive perceptions, and behavioral outcomes, most notably influencing advertising attitudes, brand perceptions, and purchase intentions (Huete-Alcocer et al., 2019).

Expertise, as a key attribute of an information source, reflects the degree to which audiences perceive it as capable of delivering accurate and relevant expertise. A highly professional information source provides audiences with reliable, in-depth knowledge, thereby fostering psychological compliance and trust (Dong et al., 2023). This perceived expertise can shape consumer attitudes positively (Liu et al., 2023). Product recommendation chatbots, for instance, rely on advanced algorithms and vast user data to analyze consumer behavior and generate tailored product suggestions. These features contribute to the perceived precision and expertise of chatbot recommendations. Moreover, many chatbots possess learning capabilities that allow them to continuously refine their algorithms based on user feedback and behavioral data, enhancing both the accuracy and trustworthiness of their suggestions (Haugeland et al., 2022). Consequently, consumers may perceive such chatbots as both professional and trustworthy, fostering more favorable attitudes toward the recommended products and increasing their purchase intentions.

Trustworthiness, another critical attribute of an information source, pertains to the perceived trustworthiness of the content provider. When facing important decisions, individuals typically seek out credible sources to obtain relevant and reliable information, which helps reduce perceived risks and uncertainty (Erdogan, 1999). Chatbots that analyze users’ historical behaviors and preferences can offer highly personalized recommendations, increasing users’ trust. In addition, the consistency and data-driven nature of chatbot recommendations—free from many human biases—further contribute to their perceived trustworthiness (Lin et al., 2020). As such, credible chatbots reduce consumers’ informational uncertainty, filling knowledge gaps and acting as reliable reference points during the decision-making process.

Interactivity refers to the extent and quality of real-time communication between the information source and the audience, thereby creating a sense of social presence (He et al., 2022). Chatbots commonly leverage advanced natural language processing (NLP) technologies to interpret and engage with natural language inputs from users (Attigeri et al., 2024). This enables them to conduct fluid and dynamic conversations that enhance perceived interactivity. Additionally, chatbots often deliver real-time feedback, which further facilitates interactive dialog (Tsai et al., 2021). This real-time responsiveness allows users to ask questions, share opinions, or request additional information, deepening their engagement. By fostering two-way communication, chatbot interactivity not only enhances users’ understanding of the products but also increases their involvement in the decision-making process (Meier et al., 2024). The more engaged users are, the more likely they are to develop favorable attitudes and make informed purchase decisions.

Customization stands as a cornerstone of product recommendation chatbot functionality within the e-commerce domain. This attribute refers to a chatbot’s ability to tailor its responses and product suggestions to fit the specific preferences and needs of individual users (Skjuve et al., 2021). Such adaptive capacity enables chatbots to serve diverse consumer groups effectively across various application scenarios. Through AI-driven analytics, chatbots swiftly interpret user data to predict interest areas and deliver personalized recommendations (Jiang et al., 2023). This tailored engagement not only enhances operational efficiency but also nurtures stronger consumer–brand relationships. Advanced customization further enables chatbots to monitor and learn from user behavior in real time, refining their ability to anticipate needs with greater accuracy (Wang C. et al., 2023; Wang L. et al., 2023; Wang X. et al., 2023). By dynamically adjusting their outputs to align with individual user profiles, chatbots elevate the quality of user interaction and overall satisfaction. Thus, AI-enabled customization enriches the consumer experience and strengthens the persuasive power of chatbot interactions, ultimately influencing user preferences and purchase intentions.

Therefore, the following hypothesis is proposed:

*H6:* Expertise has a significant positive impact on the intention of Generation Z to purchase products recommended by product recommendation chatbots.

*H7:* Interactivity has a significant positive impact on the intention of Generation Z to purchase products recommended by product recommendation chatbots.

*H8:* Trustworthiness has a significant positive impact on the intention of Generation Z to purchase products recommended by product recommendation chatbots.

*H9:* Customization has a significant positive impact on the intention of Generation Z to purchase products recommended by product recommendation chatbots.

### 3.3 Personal innovativeness

Personal innovativeness refers to an individual’s propensity to embrace novel ideas, methods, and technologies (Walley et al., 2017). It reflects one’s willingness to take risks and adapt to change. Individuals exhibiting high levels of innovativeness are typically driven to seek out experiences that are new and unconventional (Ali and Warraich, 2023). Such individuals are more inclined to explore emerging products and technologies as a means of satisfying their curiosity and pursuit of novelty (Alkawsi et al., 2021). As chatbot-mediated shopping represents a novel digital experience, it is likely to appeal to highly innovative consumers who demonstrate greater openness to technological experimentation. Furthermore, these individuals often possess a heightened capacity to adapt to and adopt new technologies (Venkatesh et al., 2012). They may find it easier to comprehend and engage with the services offered by chatbots and exhibit greater willingness to purchase chatbot-recommended products. Their receptivity to innovation enhances their ability to recognize and appreciate the convenience and benefits enabled by advanced technologies.

Accordingly, the following hypothesis is proposed:

*H10:* Personal innovativeness positively moderates the influence of personality traits on the intention of Generation Z to purchase products recommended by product recommendation chatbots.

Consumer decision-making in chatbot-assisted environments is a result of the interaction between internal psychological predispositions and external information stimuli. The Big Five personality traits serve as a theoretically grounded framework for capturing stable interindividual differences in cognition, emotion, and behavioral tendencies. These traits influence how consumers attend to, interpret, and react to external cues in digital environments. For example, traits such as openness or neuroticism may alter the way individuals perceive risk, trustworthiness, or novelty when interacting with artificial agents. On the other hand, information source characteristics—such as perceived expertise, trustworthiness, and interactivity—represent functional cues emitted by the chatbot that guide users’ evaluations of message quality and decision relevance. The decision to integrate these two dimensions in a single model stems from the recognition that behavioral outcomes such as purchase intention are not merely functions of either user traits or system attributes alone, but emerge from their interplay.

This theoretical integration enables the investigation of how personality-based perceptual filters modulate responses to chatbot-generated recommendations. It assumes that the influence of information cues is not uniform across individuals but is differentially processed depending on who the user is. In this sense, personality traits act as endogenous filters that shape the salience and interpretive meaning of chatbot characteristics. For instance, while interactivity may enhance purchase intention among extraverted users who enjoy social-like interaction, the same feature may be less effective or even distracting for individuals high in conscientiousness who prioritize efficiency and clarity.

**Figure 2:**
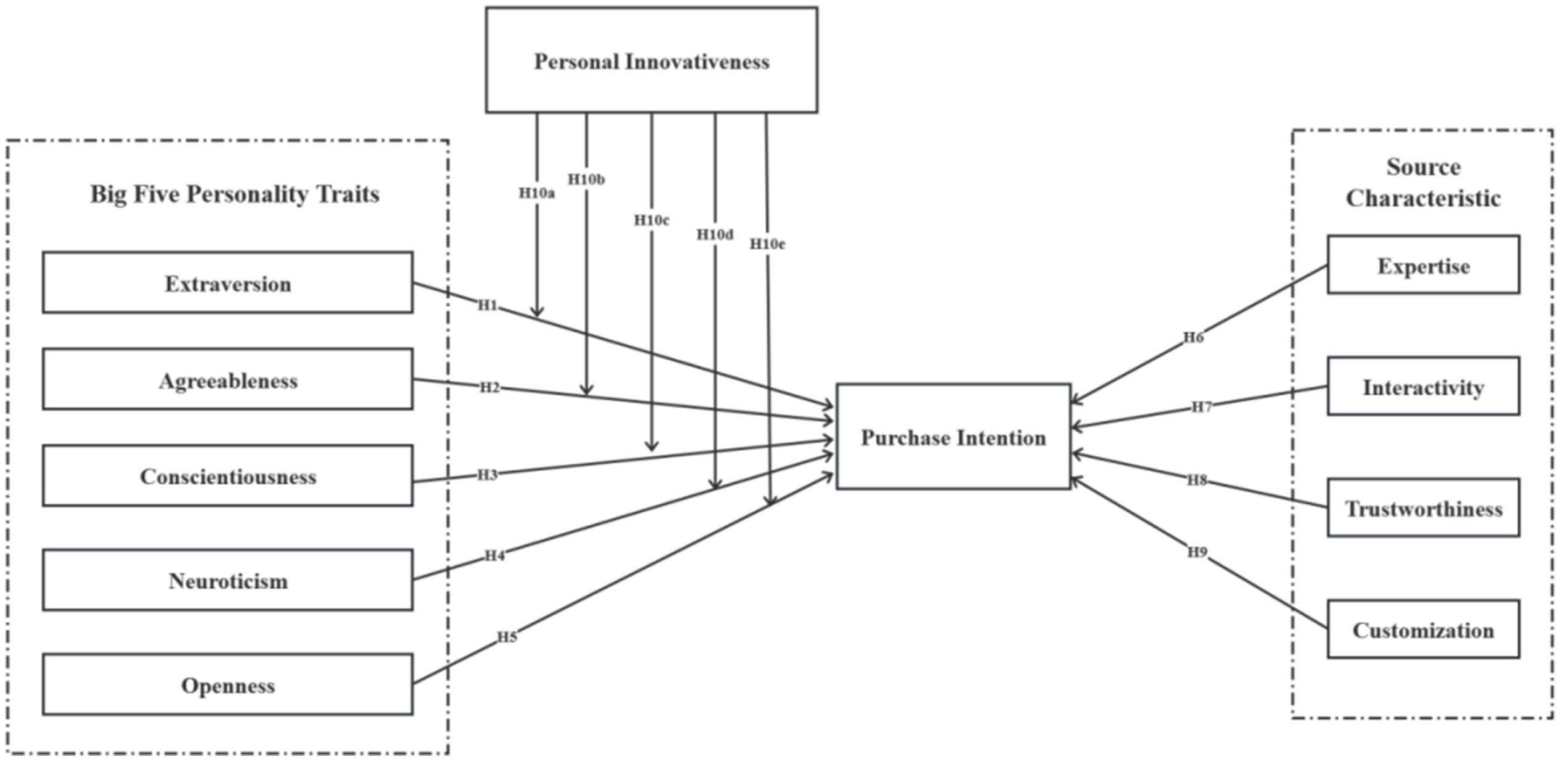
Research model.

In addition, the inclusion of personal innovativeness as a moderating variable further refines the model by accounting for variability in users’ openness to novel technologies. Even among individuals with similar personality profiles, those with higher levels of innovativeness are more likely to engage with and respond positively to chatbot-driven interaction, especially when the chatbot exhibits high trustworthiness or advanced interactive capabilities. By modeling personal innovativeness as a moderator, the framework captures the boundary conditions under which internal traits and external cues jointly translate into behavioral intentions. Therefore, the combination of personality traits, information source characteristics, and innovativeness orientation provides a comprehensive structure for understanding individual-level variability in AI-mediated consumer behavior. The research hypothesis model is shown in Figure 2.

## 4 Research methodology

### 4.1 Measurement

This study employed a structured survey questionnaire to empirically evaluate the proposed research model. To ensure the validity and reliability of the measurement, each construct in the model was operationalized using multiple items adapted from well-established scales in prior literature. These items were carefully modified to align with the specific research context. As outlined in Table 1, the measurement framework included items for purchase intention, drawn from Pavlou (2003), and dimensions of product recommendation chatbots, informed by Ohanian (1991), Gorham (1988), Yang et al. (2023), and Periaiya and Nandukrishna (2023). The chatbot-related constructs were conceptualized across four dimensions: expertise, trustworthiness, interactivity, and customization. The Big Five personality traits were measured using items based on the scale developed by Benet-Martínez and John (1998), while personal innovativeness was assessed using items primarily adapted from Agarwal and Prasad (1999). To ensure consistent interpretation of trait direction, the measurement items for Neuroticism were reverse-coded prior to analysis, as indicated in Table 1. All measurement items employed a seven-point Likert scale, ranging from 1 (“strongly disagree”) to 7 (“strongly agree”), enabling respondents to express the degree of their agreement with each statement.

**Table 1:** Measurement scales.

Variable	Items	Source
Expertise	I believe that the product recommendation chatbot I use possesses professional skills	Yang et al. (2023), Ohanian (1991), and Gorham (1988)
	I think the product recommendation chatbot I use has special skills and expertise	
	I believe the product recommendation chatbot I use is knowledgeable	
	I feel that the product recommendation chatbot I use has extensive experience in recommending products	
Trustworthiness	I believe the products recommended by the product recommendation chatbot I use are trustworthy	
	I trust the product recommendation chatbot I use	
	I believe the content recommended by the product recommendation chatbot I use is reliable	
Interactivity	I believe I have a good interactive relationship with the product recommendation chatbot I use	
	I feel that the content recommended by the product recommendation chatbot I use allows me to engage effectively	
	I think the content recommended by the product recommendation chatbot I use can pique my interest	
Customization	I believe the product recommendation chatbot shows me customized content	Periaiya and Nandukrishna (2023)
	I feel the product recommendation chatbot is tailored for my use	
	I think the products recommended by the product recommendation chatbot are tailored for my use	
Purchase intention	If given the opportunity, I plan to purchase the products recommended by the product recommendation chatbot.	Pavlou (2003)
	If given the opportunity, I predict that I will purchase the products recommended by the product recommendation chatbot in the future.	
	I am highly likely to purchase the products recommended by the product recommendation chatbot in the near future.	
Extraversion	I feel I am outgoing and sociable person	Benet-Martínez and John (1998)
	I am very talkative	
	I have an assertive personality	
	I usually generate a lot of enthusiasm	
Agreeableness	I am considerate to almost everyone	
	I like to cooperate with others	
	I am always helpful and unselfish with others	
	I have a forgiving nature	
Conscientiousness	I will do job thoroughly	
	I do things efficiently	
	I stick to my plans	
	I am a reliable person	
Neuroticism	I do not worry a lot	
	I never get tensed	
	I do not get nervous easily	
	I generally remain calm in tense situations	
Openness	I am more inventive	
	I am open to new ideas	
	I feel I have active imagination	
	I like to reflect and play with ideas	
	I love art, music and literature	
	I am a deep thinker	
	I am curious about many different things	
	I prefer to do works that is challenging	
Personal innovativeness	I believe I am ready and capable of using innovative technologies such as product purchase in product recommendation chatbots	Agarwal and Prasad (1999)
	When I hear about new information technology I would look for ways to experiment with it	
	I like to experiment with new IT products	
	Among my peers, I am usually the first to try IT products	

A seven-point Likert scale was selected because prior psychometric research shows that scales with 5–7 response categories maximize reliability, item discrimination, and respondent preference while avoiding the cognitive overload associated with longer formats (Preston and Colman, 2000; Finstad, 2010). In the context of consumer-behavior surveys, a seven-point format provides finer granularity than a five-point scale yet retains cross-cultural comparability (Dawes, 2008). Recent chatbot and technology-adoption studies have likewise adopted seven-point scales for the same reasons of sensitivity and ease of interpretation (Yang et al., 2023; Periaiya and Nandukrishna, 2023). Therefore, using a seven-point Likert scale aligns with best practice and enhances the psychometric quality of our measurements.

In addition, the questionnaire included demographic variables such as age, gender, occupation, and place of residence. Following the initial compilation of the survey instrument, the questionnaire was translated into Chinese by a native Chinese-speaking researcher to ensure linguistic and contextual appropriateness. To further enhance clarity and accuracy, five graduate students in management—each with prior experience using product recommendation chatbots—were invited to review and provide feedback on the translated version. Subsequently, the revised Chinese questionnaire was back-translated into English by a researcher with expertise in academic English to ensure semantic equivalence and consistency across both language versions.

### 4.2 Data collection and descriptive statistics

This study conducted an online questionnaire survey targeting Generation Z consumers in China (aged 16–29) to empirically examine the proposed research hypotheses. Several factors justify the focus on the Chinese context. First, China boasts an enormous online population of approximately 1.092 billion, with an internet penetration rate of 77.5%. A significant portion of these users belong to Generation Z. Second, product recommendation chatbots are widely implemented in Chinese e-commerce platforms and have become an integral part of online shopping experiences.

The questionnaire was designed and administered using Wenjuanxing,^1^ the largest and most widely utilized online survey platform in China. Wenjuanxing is frequently adopted by both domestic and international enterprises as well as academic institutions due to its robust sampling capabilities and efficient data collection infrastructure. To obtain a sufficient number of eligible respondents, a convenience sampling approach was employed. The survey link was disseminated through three major Chinese social media platforms—Sina Weibo, Tencent WeChat, and Douyin (TikTok China)—via posts, private messages, and group announcements. As an incentive, participants who completed the survey received a nominal monetary reward of 5 RMB. Prior to the main questionnaire, two mandatory screening questions were used to ensure respondent eligibility: (a) “Are you currently aged 16–29 (Generation Z)?” and (b) “Have you ever used product recommendation services provided by chatbots?” Respondents who answered “No” to either question were automatically excluded from the survey. Eligible participants then proceeded to complete the full set of measurement items covering all constructs of interest: Big Five personality traits, perceived information source characteristics (expertise, trustworthiness, interactivity, and customization), personal innovativeness, and purchase intention, along with demographic information.

A total of 683 responses were collected. After excluding questionnaires from individuals who did not meet the age criterion, had not used chatbot-based product recommendations, provided incomplete responses, completed the survey in less than 1 min, or selected the same option for all items, a final valid sample of 480 responses was retained for analysis. Descriptive statistics for the sample are summarized in Table 2. Regarding age, the respondents ranged from 16 to 29 years old, with a mean of 21.86 years and a standard deviation of 2.41. In terms of gender, 73.33% (*n* = 352) identified as male and 26.67% (*n* = 128) as female. Regarding occupation, 72.71% (*n* = 349) were students and 27.29% (*n* = 131) were employed professionals. With respect to residence, 82.29% (*n* = 395) reported living in urban areas, while 17.71% (*n* = 85) resided in rural areas.

**Table 2:** Demographic information.

Demographic measures	Count	Percentage
Gender		
Female	128	26.67%
Male	352	73.33%
Occupation		
Students	349	72.71%
Working professionals	131	27.29%
Place of living		
Urban	395	82.29%
Rural	85	17.71%

## 5 Results

### 5.1 Common method bias

Common method bias (CMB) refers to systematic, non-substantive variance that arises from measurement artifacts—such as questionnaire format, respondent characteristics, or contextual influences—rather than the constructs of interest themselves (Chang and Zhu, 2012). It represents a critical threat to the validity of empirical findings derived from self-reported survey data. To assess the presence of CMB in this study, Harman’s single-factor test was conducted following the procedure recommended by Podsakoff et al. (2003). An exploratory factor analysis was performed using SPSS 27.0. The results revealed that 11 factors had eigenvalues greater than 1, collectively accounting for 71.90% of the total variance. Notably, the first unrotated factor accounted for only 27.78% of the variance, which falls well below the commonly accepted threshold of 40%. These findings suggest that common method bias is not a significant concern in this study.

### 5.2 Results of structural equation modeling

#### 5.2.1 Assessment of measurement model

This study assessed the reliability of the measurement scale primarily through internal consistency analyses. Specifically, composite reliability (*CR*) and Cronbach’s *α* coefficients were used as indicators. Both *CR* and Cronbach’s α values exceeding the threshold of 0.70 are considered indicative of strong reliability. As shown in Table 3, all latent constructs demonstrated *CR* and *α* values above this benchmark, confirming the reliability of the measurement model.

**Table 3:** Cronbach’s α, corporate reliability and average variance extracted.

Variable	Cronbach’s alpha	Composite reliability	AVE	Items	Factor loading
Expertise	0.811	0.875	0.636	EXP1	0.772
				EXP2	0.826
				EXP3	0.841
				EXP4	0.748
Personal innovativeness	0.936	0.954	0.838	PEI1	0.914
				PEI2	0.915
				PEI3	0.909
				PEI4	0.923
Interactivity	0.822	0.889	0.729	INT1	0.770
				INT2	0.888
				INT3	0.898
Trustworthiness	0.860	0.915	0.781	TRU1	0.881
				TRU2	0.878
				TRU3	0.892
Agreeableness	0.878	0.916	0.732	AGR1	0.868
				AGR2	0.868
				AGR3	0.835
				AGR4	0.85
Customization	0.855	0.912	0.776	CUS1	0.878
				CUS2	0.903
				CUS3	0.861
Extraversion	0.86	0.905	0.703	EXT1	0.882
				EXT2	0.837
				EXT3	0.805
				EXT4	0.829
Openness	0.876	0.897	0.529	OPE1	0.877
				OPE2	0.825
				OPE3	0.818
				OPE4	0.835
				OPE5	0.764
				OPE6	0.764
				OPE7	0.876
				OPE8	0.864
Neuroticism	0.829	0.885	0.659	NEU1	0.820
				NEU2	0.850
				NEU3	0.828
				NEU4	0.745
Conscientiousness	0.946	0.961	0.860	CON1	0.930
				CON2	0.926
				CON3	0.942
				CON4	0.910
Purchase intention	0.853	0.911	0.773	PI1	0.901
				PI2	0.880
				PI3	0.856

EXP, Expertise; PEI, Personal Innovativeness; INT, Interactivity; TRU, Trustworthiness; EXT, Extraversion; CUS, Customization; AGR, Agreeableness; OPE, Openness; NEU, Neuroticism; CON, Conscientiousness; PI, Purchase Intention.

Validity was evaluated in terms of both convergent and discriminant validity. Convergent validity was assessed through factor loadings, with values above 0.70 deemed acceptable, indicating that the observed variables adequately represent the underlying latent constructs. Discriminant validity was examined using the Fornell–Larcker criterion and the Heterotrait–Monotrait ratio (*HTMT*). According to the Fornell–Larcker criterion, discriminant validity is established when the average variance extracted (*AVE*) for each construct exceeds 0.50 and is greater than the squared correlations between constructs. *HTMT* values below 0.85 (or, in some cases, 0.90) further confirm satisfactory discriminant validity by indicating that correlations within constructs are stronger than those between different constructs.

As presented in Tables 4, 5, all factor loadings met the criteria for structural validity (Fornell and Larcker, 1981), demonstrating a strong linear relationship between observed indicators and their respective latent variables, as well as sufficient explanatory power. Furthermore, to control for potential collinearity issues among predictors, variance inflation factors (*VIF*) were examined. All *VIF* values were below the critical threshold of five, indicating that multicollinearity was not a concern in this model. The multicollinearity diagnostics reported in Tables 6, 7 further support the robustness of the structural model (Neter et al., 1990).

**Table 4:** Differential validity based on HTMT method.

Variable	EXP	PEI	INT	TRU	AGR	CUS	EXT	OPE	NEU	CON	PI
EXP											
PEI	0.186										
INT	0.106	0.036									
TRU	0.636	0.169	0.034								
AGR	0.538	0.241	0.111	0.490							
CUS	0.645	0.303	0.073	0.553	0.563						
EXT	0.210	0.140	0.069	0.309	0.295	0.379					
OPE	0.696	0.216	0.094	0.613	0.590	0.601	0.332				
NEU	0.550	0.224	0.102	0.512	0.476	0.543	0.326	0.574			
CON	0.047	0.037	0.099	0.029	0.018	0.044	0.126	0.108	0.056		
PI	0.558	0.529	0.173	0.429	0.605	0.654	0.470	0.570	0.457	0.058	

EXP, Expertise; PEI, Personal Innovativeness; INT, Interactivity; TRU, Trustworthiness; EXT, Extraversion; CUS, Customization; AGR, Agreeableness; OPE, Openness; NEU, Neuroticism; CON, Conscientiousness; PI, Purchase Intention.

**Table 5:** Fornell-Larcker criterion.

Variable	EXP	PEI	INT	TRU	AGR	CUS	EXT	OPE	NEU	CON	PI
EXP	0.798										
PEI	0.171	0.915									
INT	0.087	0.023	0.854								
TRU	−0.526	−0.151	−0.024	0.884							
AGR	0.459	0.219	0.101	−0.425	0.855						
CUS	0.534	0.273	0.065	−0.475	0.488	0.881					
EXT	0.185	0.127	0.025	−0.268	0.259	0.328	0.839				
OPE	0.631	0.226	0.087	−0.585	0.575	0.558	0.308	0.727			
NEU	−0.453	−0.204	−0.086	0.437	−0.413	−0.458	−0.282	−0.547	0.812		
CON	0.042	0.026	−0.094	−0.017	−0.003	0.023	0.114	0.076	−0.042	0.927	
PI	0.477	0.474	0.156	−0.369	0.525	0.56	0.408	0.544	−0.393	0.054	0.879

EXP, Expertise; PEI, Personal Innovativeness; INT, Interactivity; TRU, Trustworthiness; EXT, Extraversion; CUS, Customization; AGR, Agreeableness; OPE, Openness; NEU, Neuroticism; CON, Conscientiousness; PI, Purchase Intention.

**Table 6:** Collinearity test results for outer model (VIF).

Items	VIF	Items	VIF	Items	VIF	Items	VIF	Items	VIF
EXP1	1.543	INT3	2.038	CUS3	1.955	OPE6	2.321	CON4	3.979
EXP2	1.770	TRU1	2.169	EXT1	2.282	OPE7	1.879	PI1	2.363
EXP3	1.749	TRU2	2.175	EXT2	2.084	OPE8	1.474	PI2	2.114
EXP4	1.614	TRU3	2.182	EXT3	1.836	NEU1	1.800	PI3	1.949
PEI1	3.578	AGR1	2.396	EXT4	1.933	NEU2	1.977	PEI × OPE	1.000
PEI2	3.404	AGR2	2.423	OPE1	3.067	NEU3	1.838	PEI × CON	1.000
PEI3	3.384	AGR3	2.028	OPE2	2.548	NEU4	1.679	PEI × NEU	1.000
PEI4	4.101	AGR4	2.108	OPE3	2.232	CON1	4.112	PEI × EXT	1.000
INT1	1.738	CUS1	2.159	OPE4	2.786	CON2	3.990	PEI × AGR	1.000
INT2	1.846	CUS2	2.401	OPE5	2.492	CON3	4.445		

EXP, Expertise; PEI, Personal Innovativeness; INT, Interactivity; TRU, Trustworthiness; EXT, Extraversion; CUS, Customization; AGR, Agreeableness; OPE, Openness; NEU, Neuroticism; CON, Conscientiousness; PI, Purchase Intention.

**Table 7:** Collinearity test results for inner model (VIF).

Variable	PI
PI	
EXP	1.956
PEI	1.180
INT	1.036
TRU	1.733
AGR	1.681
CUS	1.817
EXT	1.208
OPE	2.510
NEU	1.577
CON	1.045
PI × AGR	1.881
PI × EXT	1.298
PI × CON	1.107
PI × NEU	2.461
PI × OPE	3.126

#### 5.2.2 Assessment of structural model

The predictive validity of the research model primarily hinges on the explanatory power of the endogenous variables. The coefficient of determination (*R^2^*) and effect size (*f^2^*) serve as the principal indicators of predictive strength, with *f*^2^ values of 0.020, 0.150, and 0.350 denoting small, medium, and large effect sizes, respectively. The results of the structural model are presented in Table 8 and Supplementary Figure 1.

**Table 8:** A summary of the PLS path analysis.

PLS path	Path coefficient	T statistics	*p*-value	95% confidence interval	
				Lower bound	Upper bound
Extraversion → purchase intention	0.216	4.515	0.000	0.116	0.306
Agreeableness → purchase intention	0.175	3.504	0.000	0.078	0.275
Conscientiousness → purchase intention	0.020	0.708	0.479	−0.039	0.074
Neuroticism → purchase intention	0.021	0.440	0.660	−0.078	0.116
Openness → purchase intention	0.149	2.612	0.009	0.042	0.265
Expertise → purchase intention	0.123	2.513	0.012	0.028	0.220
Interactivity → purchase intention	0.097	3.011	0.003	0.032	0.161
Trustworthiness → purchase intention	0.056	1.142	0.253	−0.049	0.148
Customization → purchase intention	0.194	2.658	0.008	0.044	0.335
Personal innovativeness → purchase Intention	0.329	6.272	0.000	0.221	0.425
Personal innovativeness × extraversion → purchase intention	0.104	1.966	0.045	0.020	0.223
Personal innovativeness × agreeableness → purchase intention	0.074	1.868	0.062	−0.007	0.152
Personal innovativeness × conscientiousness → purchase intention	−0.021	0.680	0.496	−0.073	0.045
Personal innovativeness × neuroticism → purchase intention	0.074	1.436	0.151	−0.031	0.172
Personal innovativeness × openness → purchase intention	0.018	0.380	0.704	−0.083	0.105

Extraversion demonstrates a significant positive effect on purchase intention (*β* = 0.216, *p* < 0.001), with an effect size of 0.094, indicating a small effect. Agreeableness also shows a significant positive relationship with purchase intention (*β* = 0.175, *p* < 0.001), but with a smaller effect size of 0.044. Neither conscientiousness (*β* = 0.020, *p* > 0.05) nor neuroticism (*β* = 0.021, *p* > 0.05) exert a significant impact on purchase intention. Openness is positively associated with purchase intention (*β* = 0.149, *p* < 0.01), with an effect size of 0.022, also reflecting a small effect.

Among the information source characteristic, expertise (*β* = 0.123, *p* < 0.05), interactivity (*β* = 0.097, *p* < 0.01), and customization (*β* = 0.194, *p* < 0.01) all have significant positive effects on purchase intention, with corresponding effect sizes of 0.020, 0.022, and 0.050, respectively—each indicating small effects. Trustworthiness does not exhibit a statistically significant impact (*β* = 0.056, *p* > 0.05).

Personal innovativeness shows a significant positive influence on purchase intention (*β* = 0.329, *p* < 0.001), with an effect size of 0.223, suggesting a moderate effect. Additionally, the interaction between personal innovativeness and extraversion is significant (*β* = 0.104, *p* < 0.05), with an effect size of 0.031. To further examine this moderation effect, Process Macros (Model 1) was employed. As illustrated in Supplementary Figure 2, extraversion has no significant impact on purchase intention at low levels of personal innovativeness (*β* = 0.120, *p* > 0.05). At moderate levels of innovativeness, extraversion shows a significant positive effect (*β* = 0.234, *p* < 0.001), which intensifies at high levels (*β* = 0.358, *p* < 0.001). These findings suggest that the positive influence of extraversion on purchase intention becomes stronger as personal innovativeness increases. In contrast, the interaction terms between personal innovativeness and agreeableness (*β* = 0.074, *p* > 0.05), conscientiousness (*β* = 0.021, *p* > 0.05), neuroticism (*β* = 0.074, *p* > 0.05), and openness (*β* = 0.018, *p* > 0.05) are not statistically significant.

Overall, the model accounts for 58.80% of the variance in Generation Z’s purchase intention regarding chatbot-recommended products. Furthermore, the blindfolding procedure was applied to evaluate the *Q*^2^-values of the five endogenous constructs. Since all *Q*^2^-values exceeded zero, the model demonstrates satisfactory predictive relevance. Specifically, the *Q*^2^-value for purchase intention (*Q*^2^ = 0.428) confirms the model’s reliable predictive capability for the endogenous outcome variables.

### 5.3 ANN results

In recent years, the reliability of structural equation modeling (SEM) has been increasingly questioned due to its inherent assumption of linear and compensatory relationships between constructs. This assumption may oversimplify the complex, multifactorial nature of decision-making processes. Given the exploratory nature of our research domain—which is still emerging and underexplored—a more robust and complementary analytical approach is necessary to validate and extend SEM findings. Accordingly, this study developed an artificial neural network (ANN) model based on the backpropagation (BP) algorithm, where extraversion (EXT), agreeableness (AGR), openness (OPE), expertise (EXP), interactivity (INT), customization (CUS), and personal innovativeness (PEI) were used as input variables, and purchase intention (PI) as the output variable.

The BP neural network minimizes the squared error between predicted and actual values using gradient descent to iteratively update weights and thresholds, optimizing the alignment between predicted outputs and expected outcomes (Denoeux and Lengellé, 1993). Table 9 presents the predictive performance of the ANN model.

**Table 9:** Accuracy values for neural network.

Neural network	Input: EXT, AGR, OPE, EXP, INT, CUS, PEI Output: PI	
	Training (%)	Testing (%)
ANN1	83.480	81.663
ANN2	80.467	80.364
ANN3	83.610	76.042
ANN4	79.303	73.415
ANN5	81.116	82.692
ANN6	80.795	80.272
ANN7	79.253	85.227
ANN8	78.355	80.167
ANN9	83.183	83.785
ANN10	85.127	78.966
Mean	81.469	80.259
SD	2.257	3.517

EXP, Expertise; PEI, Personal Innovativeness; INT, Interactivity; EXT, Extraversion; CUS, Customization; AGR, Agreeableness; OPE, Openness; PI, Purchase Intention.

To examine potential nonlinear relationships within the model, we compared the predictive performance of PLS-SEM and ANN. As recommended by Lau et al. (2021), if the ANN demonstrates superior goodness of fit relative to the linear model, this implies the presence of nonlinear patterns. The average prediction accuracy of the ANN across training and test sets ranged from 73.415 to 85.127%, with an overall average of 80.864%. In contrast, the linear PLS-SEM model accounted for 58.80% of the variance in the outcome variable (see Supplementary Figure 2). These results suggest that the nonlinear BP neural network provides a better fit to the data, thereby confirming the existence of nonlinear relationships among variables in the conceptual model.

Subsequently, a sensitivity analysis was conducted using the permutation method to rank the relative importance of each input variable. As shown in Table 10 and Supplementary Figure 3, customization (CUS, 0.217) emerged as the most influential predictor of purchase intention in the ANN model, followed closely by personal innovativeness (PEI, 0.213). These findings underscore the central role of customization in shaping Generation Z consumers’ purchasing behavior in chatbot interactions. Generative AI technologies underpinning advanced customization capabilities can accurately infer and adapt to user preferences, thereby optimizing user experiences. Through tailored recommendations, content delivery, and real-time feedback, AI systems elevate user engagement and facilitate seamless decision-making, ultimately increasing purchase likelihood among digitally savvy Generation Z users. Notably, individuals with high personal innovativeness are more open to adopting new technologies, including product recommendation chatbots, and are thus more likely to act on the product suggestions provided.

**Table 10:** Sensitivity analysis.

ANN	Input: EXT, AGR, OPE, EXP, INT, CUS, PEI Output: PI						
	EXT	AGR	CUS	OPE	EXP	INT	PEI
ANN1	0.313	0.032	0.192	0.055	0.035	0.154	0.221
ANN2	0.298	0.054	0.316	0.134	0.035	0.005	0.159
ANN3	0.227	0.194	0.141	0.039	0.148	0.022	0.230
ANN4	0.190	0.255	0.163	0.092	0.034	0.029	0.237
ANN5	0.180	0.010	0.299	0.008	0.149	0.169	0.186
ANN6	0.166	0.028	0.233	0.059	0.137	0.138	0.240
ANN7	0.027	0.259	0.196	0.268	0.078	0.061	0.112
ANN8	0.057	0.286	0.221	0.160	0.006	0.045	0.225
ANN9	0.254	0.171	0.201	0.071	0.008	0.090	0.206
ANN10	0.112	0.037	0.210	0.036	0.204	0.082	0.319
ARI	0.182	0.133	0.217	0.092	0.083	0.080	0.213
NI(%)	83.87%	61.29%	100.00%	42.40%	38.25%	36.87%	98.16%

EXP, Expertise; PEI, Personal Innovativeness; INT, Interactivity; EXT, Extraversion; CUS, Customization; AGR, Agreeableness; OPE, Openness; PI, Purchase Intention; ARI, ANN Relative importance; NI, Normalized Importance.

According to Table 11, in the PLS-SEM analysis, personal innovativeness was identified as the most influential psychological trait affecting purchase intention, while customization was ranked third among the chatbot features. In contrast, the ANN analysis identified customization as the most critical predictor, with personal innovativeness ranking second. This divergence arises from the different assumptions underlying the two models. The linear PLS-SEM assumes additive and independent effects of predictors, potentially underestimating the interactive or synergistic effects of features such as customization. In such models, personal innovativeness may appear to dominate as a stable, direct influence on behavior, while customization’s role is diluted.

**Table 11:** Comparison between PLS-SEM and ANN results.

PLS path	Original sample (O)/path coefficient	ANN results: (average relative importance)	Ranking (PLS-SEM) (based on path coefficient)	Ranking (ANN) (based on Average relative importance)	Remark
Input: EXT, AGR, OPE, EXP, INT, CUS, PEI Output: PI					
EXT → PI	0.216	0.182	2	3	Not match
AGR → PI	0.175	0.133	4	4	
OPE → PI	0.149	0.092	5	5	
EXP → PI	0.123	0.083	6	6	
INT → PI	0.097	0.080	7	7	
CUS → PI	0.194	0.217	3	1	
PEI → PI	0.329	0.213	1	2	

EXP, Expertise; PEI, Personal Innovativeness; INT, Interactivity; EXT, Extraversion; CUS, Customization; AGR, Agreeableness; OPE, Openness; PI, Purchase Intention; ARI, ANN Relative importance; NI, Normalized Importance.

By contrast, ANN is capable of capturing complex, nonlinear interactions and interdependencies among predictors. As a result, the nonlinear model identifies customization as the most critical feature, reflecting its intricate and dynamic relationship with purchase behavior—particularly when moderated by users’ levels of personal innovativeness. Thus, the superior explanatory power of customization in the ANN model highlights its central role in influencing Generation Z’s purchasing decisions in chatbot-driven e-commerce environments.

Furthermore, when comparing the linear and nonlinear models, the ranking of most predictors remained consistent, with noticeable changes only observed in customization (CUS), personal innovativeness (PEI), and extraversion (EXT). This consistency reinforces the overall stability and robustness of the model’s predictive structure across analytical approaches. The application of ANN offers enhanced precision in capturing nonlinear dynamics and deeper insights into the complex mechanisms driving Generation Z’s purchasing behavior in response to chatbot recommendations.

### 5.4 Results of NCA

After identifying the relative importance of relationships among variables within the research model, the next step involves assessing their necessity. If all relationships are deemed necessary, their *p*-values should be less than 0.05, as suggested by Dul et al. (2020). This implies that each exogenous construct in the model serves as a necessary condition for the occurrence of its respective outcome. Necessary Condition Analysis (NCA) provides a suitable framework for this assessment by evaluating the extent to which a condition must be present for an outcome to occur.

Depending on the nature of the variables, NCA employs two main upper-bound analytical techniques: Ceiling Envelopment (CE) for binary or discrete variables with fewer than five levels, and Ceiling Regression (CR) for discrete or continuous variables with five or more levels. Given that the variables in this study are predominantly continuous or multi-level discrete, CR is more appropriate and is therefore the primary method used for interpretation.

NCA evaluates necessity based on two key indicators: the necessity effect size (*d*) and the significance level derived from Monte Carlo permutation testing. The effect size *d* ranges from 0 to 1, with higher values indicating a greater degree of necessity. A statistically significant *p*-value (*p* ≤ 0.05) confirms that the predictor is a necessary condition for the outcome variable. As shown in Supplementary Table 1, Agreeableness, Openness, Expertise, Interactivity, and Customization all exhibit significant necessity effects for purchase intention. In contrast, Extraversion shows a CR effect size of 0.042 with a *p*-value of 0.376, suggesting it is not a necessary condition (*p* > 0.05). Similarly, Personal Innovativeness has a CR effect size of 0.014 with a *p*-value of 0.336, indicating it does not qualify as a necessary condition either.

To further explore how varying levels of conditional factors influence purchase intention, a bottleneck analysis was conducted (see Supplementary Table 2). This analysis determines the minimum level each conditional factor must attain to achieve specific thresholds of purchase intention, ranging across the observed spectrum.

The results indicate that to achieve a 50% purchase intention, the required levels for Agreeableness, Openness, Expertise, and Interactivity are 25.00, 28.57, 25.00, and 23.81%, respectively, while Customization remains nonessential (0%). At the 80% purchase intention level, the required levels rise to 28.57% for Openness, 35.71% for Expertise, 23.81% for Interactivity, and 19.04% for Customization, while Agreeableness remains constant at 25%. At the highest threshold (80%), the required levels for Openness, Expertise, Interactivity, and Customization further increase to 37.50, 50.00, 33.33, and 23.81%, respectively, with Agreeableness still unchanged at 25%.

These findings suggest that at lower thresholds of purchase intention, Agreeableness, Openness, Expertise, and Interactivity function as more critical necessary conditions, whereas the role of Customization is minimal. However, as the desired level of purchase intention increases, the importance of Openness, Expertise, Interactivity, and Customization as necessary conditions becomes more pronounced. Notably, the required level of Agreeableness remains constant across all thresholds, indicating its consistent role as a foundational necessity in driving purchase intention.

## 6 Discussion

Over the past two decades, the rapid advancement of technology has profoundly transformed daily life. The continuous evolution of artificial intelligence (AI) has brought about substantial changes in education, consumption, social interaction, and culture. Recently, the emergence of chatbots has begun to exert a significant influence on consumer behavior. Generation Z, as the primary users of digital technology, plays a crucial role in shaping consumer behavior through interactions with product recommendation chatbots. Accordingly, this study investigates the key determinants—namely, personality traits and chatbot characteristics—that influence Generation Z consumers’ purchase of chatbot-recommended products.

The structural equation modeling (SEM) results reveal that the Big Five personality traits significantly predict the purchase intentions of Generation Z consumers. Extraversion, reflecting social activity, enthusiasm, and openness, was positively associated with engagement. Highly extraverted consumers are typically more responsive to social influence, including peer, influencer, and chatbot-based recommendations (Marengo et al., 2020). Their comfort with new technologies enhances their receptivity to chatbot interactions and increases their likelihood of accepting suggested products (Gan, 2016). Agreeableness, which encompasses cooperativeness, trust, empathy, and friendliness, was also positively associated with purchase intention. Consumers high in agreeableness are more inclined to trust recommendation systems, perceiving chatbot suggestions as supportive and well-intentioned (Sowmya et al., 2023). These consumers tend to appreciate personalized services, and chatbot customization addresses their desire for tailored experiences (Fazli-Salehi et al., 2021). Openness, which captures creativity, curiosity, and receptiveness to novelty, positively influences consumers’ willingness to explore and adopt new products and technologies (Tucaković and Nedeljković, 2022; Duong, 2021). Consumers high in openness are more likely to try novel or unconventional products suggested by chatbots. In contrast, conscientiousness and neuroticism did not show significant effects on purchase intention. One explanation is that conscientious consumers prefer transparent and structured decision-making processes and may resist opaque or dynamic algorithmic recommendations (Nordheim et al., 2019). Meanwhile, neurotic consumers often exhibit anxiety and mistrust toward unfamiliar technologies, including AI agents, leading to lower adoption intentions (Wang C. et al., 2023; Wang L. et al., 2023; Wang X. et al., 2023).

Personal innovativeness, defined as the degree to which individuals are open to new technologies (Agarwal and Prasad, 1999), emerged as a strong predictor of chatbot-driven purchases. Highly innovative consumers tend to explore and embrace new tools such as chatbots (Ali and Warraich, 2023). Moreover, personal innovativeness positively moderates the effect of extraversion on purchase intention, suggesting that highly innovative and extraverted consumers engage more frequently and meaningfully with chatbots, increasing their likelihood of purchasing.

The characteristics of product recommendation chatbots also play a crucial role. Expertise, interactivity, and customization significantly influence purchase intention. Consumers are more inclined to trust recommendations from chatbots that demonstrate professional knowledge, offer human-like interaction, and tailor content based on preferences and behavior (Nordheim et al., 2019; Shin and Choi, 2021; Orden-Mejía et al., 2023; Periaiya and Nandukrishna, 2023). Customization, in particular, allows users to feel greater control over the shopping experience, fostering engagement and purchase intent. Conversely, trustworthiness did not significantly affect purchase intention. This finding deviates from traditional source trustworthiness theory but aligns with newer perspectives indicating that Generation Z emphasizes functional benefits—such as speed, usability, and fit—over institutional trust (Reinikainen et al., 2020; Lajante et al., 2023).

Artificial neural network (ANN) analysis provided further insights. While SEM identified personal innovativeness as the most influential factor and customization as third, ANN results indicated that customization had the strongest predictive power, followed by personal innovativeness. This divergence underscores the importance of modeling nonlinear relationships. ANN’s capacity to capture complex interactions reveals that consumers assign greater value to highly personalized shopping experiences in real-world settings (Wang C. et al., 2023; Wang L. et al., 2023; Wang X. et al., 2023; Chen and Wu, 2024). Thus, ANN complements the linear assumptions of SEM by providing a more nuanced understanding of consumer behavior.

Lastly, necessary condition analysis (NCA) revealed that agreeableness, openness, expertise, interactivity, and customization are essential for purchase intention—confirming their roles across all analytical methods. Interestingly, extraversion and personal innovativeness, though influential, were not necessary conditions. While extraverted consumers may be more socially inclined, introverts—who prefer solitude or small-group communication—can still exhibit strong purchase intent when supported by personalized chatbot recommendations (Lim et al., 2019). Similarly, although personal innovativeness enhances adoption, modern chatbot systems can accommodate a wide range of user preferences, diminishing the necessity of high innovativeness for generating purchase behavior (Jackson et al., 2013).

Together, these findings highlight the multifaceted psychological and technological determinants of Generation Z’s purchase decisions in chatbot-assisted e-commerce environments. By integrating personality psychology with AI-enabled commerce, this study contributes both theoretical clarity and practical implications for chatbot design and personalization strategies.

## 7 Conclusion

### 7.1 Theoretical contributions

This study offers several key theoretical contributions to the existing literature on consumer behavior in AI-mediated e-commerce environments, particularly in the context of Generation Z’s interactions with product recommendation chatbots. By integrating the Big Five personality traits with the attributes of product recommendation chatbots, this research provides a more comprehensive understanding of how personality traits and chatbot characteristics jointly influence purchasing decisions. Our findings contribute to the theoretical understanding of both personality psychology and digital consumer behavior, bridging gaps in previous research and offering new insights into the complex mechanisms that drive Generation Z’s responses to chatbot-based product recommendations.

One of the primary contributions of this study is its exploration of the intersection between consumer personality traits and chatbot attributes, which has not been sufficiently examined in prior research. While existing studies have addressed the role of personality in technology adoption (Duong, 2021; Liu et al., 2024) and the influence of chatbot features on user acceptance (Zarouali et al., 2018), little attention has been given to how these factors interact. Our study integrates these two perspectives, examining how the Big Five personality traits—extraversion, agreeableness, conscientiousness, neuroticism, and openness—interact with key features of chatbots, such as expertise, trustworthiness, interactivity, and customization, to shape Generation Z’s purchase intentions. This integration bridges an important gap in the literature by offering a multi-faceted model that explains how consumers’ psychological traits and the functional features of chatbots jointly influence purchasing behavior. For example, while extraverted individuals are more likely to engage with interactive chatbots and derive greater benefit from personalized recommendations, conscientious individuals tend to prefer chatbots that offer structured and reliable information (Huang et al., 2024; Moisescu et al., 2025). This nuanced understanding is a significant departure from prior research, which often treats chatbot features and personality traits as independent predictors.

Another significant contribution of this study is its empirical investigation of how individual personality traits, particularly extraversion, influence Generation Z’s responses to product recommendations. While the role of personality traits in general technology adoption has been well established (Svendsen et al., 2013), this study goes a step further by demonstrating that certain personality traits, such as extraversion, play a particularly strong role in shaping consumer behavior in the context of AI-mediated interactions. Extraverted individuals are more likely to engage with chatbots, appreciating their interactive and socially engaging nature, which in turn increases their purchase intentions. This finding is consistent with prior research that suggests that extraverted individuals enjoy social interaction and seek external sources of stimulation (Sowmya et al., 2023). By linking personality psychology with consumer behavior theory, this study introduces a novel framework that not only extends existing research on technology adoption but also provides a new understanding of how individual personality differences influence purchasing decisions in AI-driven environments.

Furthermore, this research contributes to the literature on chatbot functionality by identifying which specific features of chatbots are most influential in shaping consumer behavior. While previous studies have generally acknowledged the importance of chatbot attributes such as expertise, trustworthiness, and interactivity (Gursoy et al., 2022), they often fail to specify which attributes are most effective in driving user acceptance and purchase intentions. Our findings reveal that expertise, interactivity, and customization significantly affect Generation Z’s purchasing behavior, providing a more granular understanding of how these features contribute to chatbot effectiveness. These results extend the current literature by pinpointing the specific functional characteristics that enhance chatbot performance, offering practical implications for both academics and practitioners. For example, personalized chatbot recommendations that align with user preferences are found to be particularly influential in increasing purchase intention among Generation Z consumers, especially those high in openness (Yang et al., 2023).

In addition, the introduction of personal innovativeness as a moderating variable offers a unique contribution to the understanding of consumer behavior in AI-mediated environments. While personal innovativeness has been studied in the context of general technology adoption (Agarwal and Prasad, 1999), it has not been extensively explored in relation to chatbot interactions. By showing that personal innovativeness moderates the relationship between personality traits and purchase intention, this study provides a more dynamic model of consumer behavior, highlighting that consumers who are more open to new technologies are more likely to respond positively to chatbot recommendations. This insight provides a deeper understanding of the variability in user responses to AI-driven interactions and helps to refine the theoretical models of technology acceptance by incorporating individual differences in openness to innovation.

Lastly, the methodological approach used in this study is also a significant contribution to the field. By combining Structural Equation Modeling (SEM), Artificial Neural Networks (ANN), and Necessary Condition Analysis (NCA), this research offers a hybrid approach that captures both linear and nonlinear relationships in consumer decision-making. SEM identifies significant predictors based on linear relationships, while ANN allows for the examination of nonlinear associations and ranks variables according to their predictive power. NCA further identifies essential conditions for the occurrence of purchase behavior, adding an additional layer of understanding to the decision-making process. This integrated methodology enhances both the explanatory power and robustness of the findings, providing a comprehensive framework for future research on consumer behavior in AI-driven environments. It also offers a more refined approach to understanding the complex interactions that drive purchasing decisions in digital commerce (Chen and Wu, 2024; Zhu et al., 2025).

### 7.2 Practical implications

The findings of this study hold practical implications for e-commerce platforms aiming to influence Generation Z consumers’ purchasing behaviors through their interactions with product recommendation chatbots. First, chatbot developers should prioritize emphasizing the personalized benefits of chatbot technology to attract young consumers. Specifically, product recommendation chatbots should highlight their ability to deliver tailored recommendations based on users’ past behaviors, preferences, and feedback. To this end, it is essential for managers to continuously upgrade and fine-tune the natural language processing and machine learning capabilities of these systems to ensure they effectively address individual needs.

Second, the study underscores the importance of considering individual differences when designing chatbot-based marketing strategies for younger consumers. A nuanced understanding of users’ big five personality traits can enable managers to craft more resonant and effective marketing content. Prior to developing targeted strategies, it is advisable for managers to conduct detailed assessments of personality profiles within their intended user base. Aligning product recommendations with these personality dimensions can enhance the perceived relevance of the offerings, thereby improving user satisfaction and increasing purchase intention.

Moreover, enhancing the expertise and interactivity of chatbots should be a key managerial priority. Product recommendations that demonstrate domain-specific expertise—such as detailed knowledge of product features, user reviews, and current market trends—can bolster user confidence in the chatbot’s reliability. Additionally, chatbots that recall users’ prior interactions and preferences reflect a deeper understanding of their individual journeys. By engaging users in a friendly, courteous, and human-like manner, chatbots can evolve from functional tools into relatable digital companions, increasing user willingness to interact.

Finally, to appeal to consumers with high personal innovativeness, managers should ensure that product recommendation chatbots embody a degree of technological novelty. Innovative interaction designs—such as immersive interfaces and novel features—can enhance user engagement. Integration of emerging technologies such as voice recognition and virtual reality should be considered to elevate the user experience and stimulate curiosity. In addition, digital platform managers should explore new features that cater to diverse user needs and enhance enjoyment, such as enabling real-time social interaction or gamified recommendation environments.

### 7.3 Limitation and future research

While this study offers theoretical insights and practical implications, it is subject to several limitations that should be acknowledged. Firstly, the sample is geographically and demographically restricted, focusing solely on generation Z consumers in China. Within this group, the majority of respondents were male (73.33%) and students (72.71%), introducing a gender and student bias that may limit the generalizability of the findings to broader populations. This sample composition may have influenced certain trait-based patterns (e.g., extraversion or openness) and response tendencies, which could differ in more gender-balanced or occupationally diverse populations.

Secondly, the reliance on self-reported questionnaire data may only capture participants’ subjective perceptions and intended behaviors, rather than actual purchasing behavior. Although constructs like purchase intention and chatbot experience were well operationalized, the study does not account for potential discrepancies between intention and behavior, especially in real-life decision-making environments.

Thirdly, the cross-sectional nature of the data collection limits our ability to observe temporal changes in consumer behavior. For instance, the influence of personalization or trustworthiness on purchase intention may evolve as users become more familiar with chatbots or as chatbot technology advances.

To address these limitations, future studies could diversify their sample demographics, including participants from different age brackets, occupations, and cultural contexts, to enable comparative cross-cultural analysis. Furthermore, incorporating experimental or neuroscience methods such as EEG or fMRI could provide deeper insight into the cognitive and affective processes underlying generation Z’s responses to chatbot interactions. Finally, longitudinal research designs would allow scholars to explore how repeated exposure to chatbots and evolving user preferences affect the dynamics of purchase behavior over time.

## Data Availability

The original contributions presented in the study are publicly available. This data can be found here: https://www.jianguoyun.com/p/DUN6uAwQ_t_QDRiz9oMGIAA. Further inquiries can be directed to the corresponding author.

## Footnotes

^1^ https://www.wjx.cn/
