# Supplementary material for: Chatbot-aided product purchases among Generation Z: the role of personality traits
Source: Front Psychol. 2025 Aug 29;16:1454197. doi: 10.3389/fpsyg.2025.1454197 (PMC12425985; doi:10.3389/fpsyg.2025.1454197)
Supplement: Supplementary file 1 [file Table_1.docx]

Supplementary Material

Chatbot-Aided Product Purchases Among Generation Z: The Role of Personality Traits

# Supplementary Figures and Tables

## Supplementary Figures

| 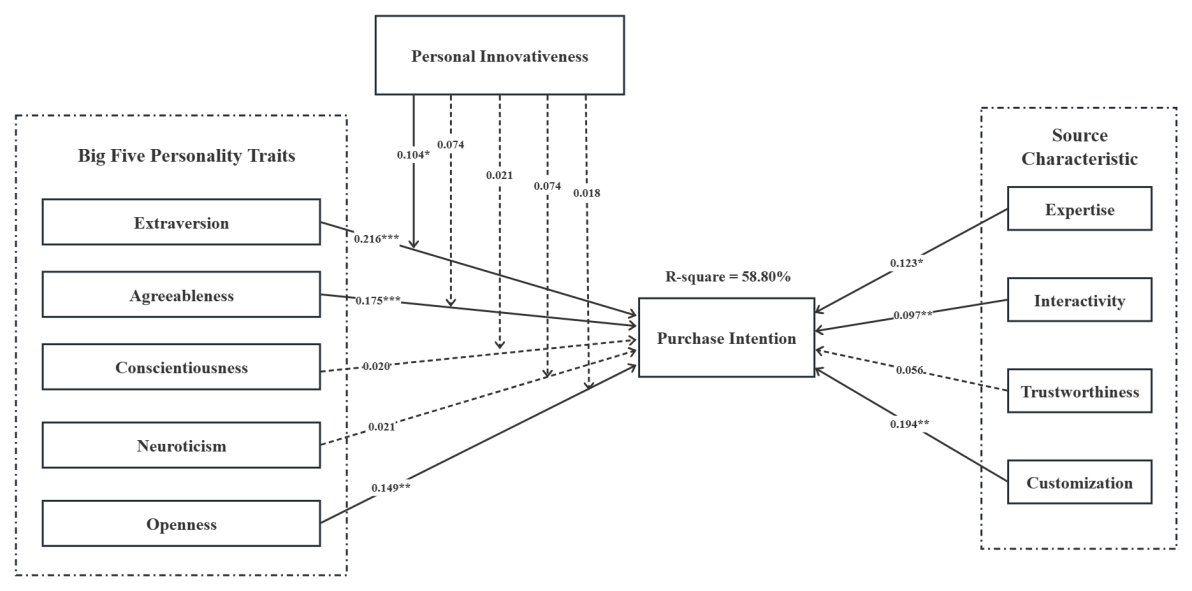  **Supplementary Figure 1.** PLS results of structural model  Note: ***p < 0.001，**p < 0.01，*p < 0.05. |
| --- |

| 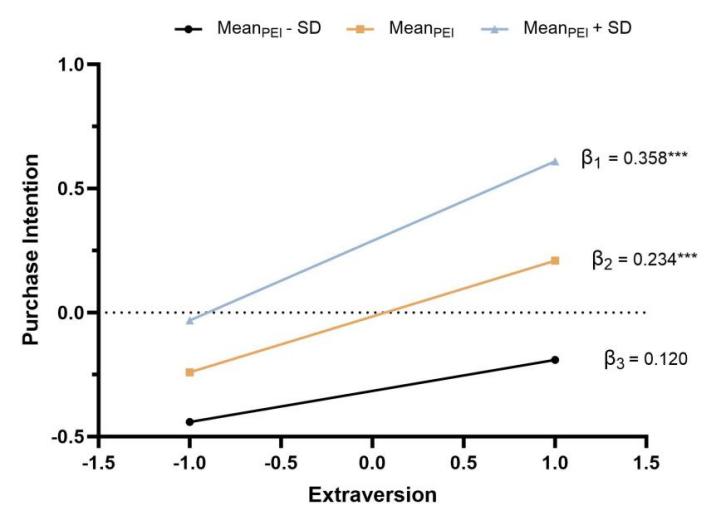  **Supplementary Figure 2.** PLS results of structural model  Note: PEI = Personal Innovativeness; β1 is the effect size of MeanPEI - SD, β2 is the effect size of MeanPEI, β3 is the effect size of MeanPEI + SD；***p < 0.001，**p < 0.01，*p < 0.05. |
| --- |
| 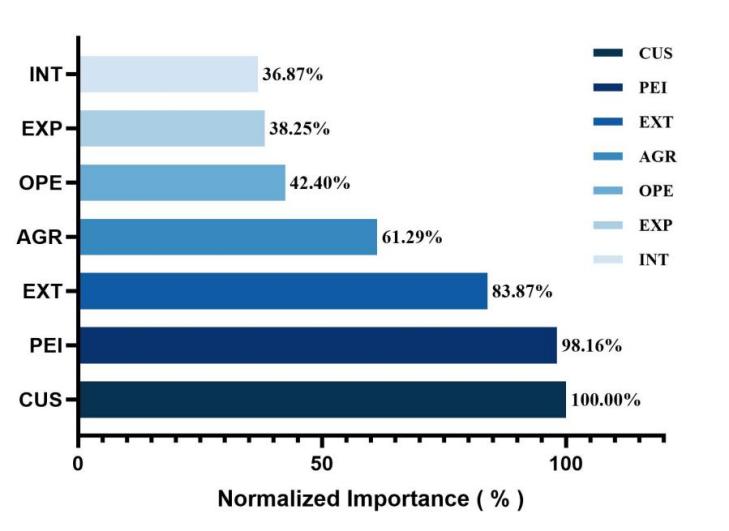  **Supplementary Figure 3.** Normalized importance of ANN  Note: EXP = Expertise, PEI = Personal Innovativeness, INT = Interactivity, EXT = Extraversion, CUS = Customization, AGR = Agreeableness, OPE = Openness, PI = Purchase Intention. |

## Supplementary Tables

**Supplementary Table 1.** Results of NCA.

| Conditional Variable | Method | Accuracy | Effect Size | p-value |
| --- | --- | --- | --- | --- |
| Extraversion | CE | 100.00% | 0.083 | 0.112 |
|  | CR | 99.79% | 0.042 | 0.376 |
| Agreeableness | CE | 100.00% | 0.083 | 0.001 |
|  | CR | 99.79% | 0.042 | 0.034 |
| Openness | CE | 100.00% | 0.119 | 0.000 |
|  | CR | 99.38% | 0.095 | 0.000 |
| Expertise | CE | 100.00% | 0.171 | 0.000 |
|  | CR | 99.38% | 0.144 | 0.000 |
| Interactivity | CE | 100.00% | 0.044 | 0.060 |
|  | CR | 99.38% | 0.059 | 0.0416 |
| Customization | CE | 100.00% | 0.022 | 0.000 |
|  | CR | 99.59% | 0.112 | 0.002 |
| Personal Innovativeness | CE | 100.00% | 0.155 | 0.357 |
|  | CR | 99.79% | 0.014 | 0.336 |

**Supplementary Table 2.** Bottleneck level analysis results.

| Purchase  Intention | Extraversion | Agreeableness | Openness | Expertise | Interactivity | Customization | Personal Innovativeness |
| --- | --- | --- | --- | --- | --- | --- | --- |
| 0.00% | NN | NN | NN | NN | NN | NN | NN |
| 10.00% | 21.43% | NN | NN | NN | NN | NN | NN |
| 20.00% | 21.43% | NN | 16.07% | 21.43% | NN | NN | 25.00% |
| 30.00% | 21.43% | NN | 16.07% | 21.43% | NN | NN | 25.00% |
| 40.00% | 21.43% | 25.00% | 16.07% | 25.00% | 23.81% | NN | 25.00% |
| 50.00% | 21.43% | 25.00% | 28.57% | 25.00% | 23.81% | NN | 25.00% |
| 60.00% | 21.43% | 25.00% | 28.57% | 35.71% | 23.81% | NN | 25.00% |
| 70.00% | 21.43% | 25.00% | 28.57% | 35.71% | 23.81% | NN | 35.71% |
| 80.00% | 21.43% | 25.00% | 28.57% | 35.71% | 23.81% | 19.04% | 35.71% |
| 90.00% | 21.43% | 25.00% | 37.50% | 50.00% | 33.33% | 23.81% | 35.71% |
| 100.00% | 21.43% | 25.00% | 37.50% | 50.00% | 33.33% | 23.81% | 35.71% |
